# Circular RNA hsa_circ_0006401 promotes proliferation and metastasis in colorectal carcinoma

**DOI:** 10.1038/s41419-021-03714-8

**Published:** 2021-05-04

**Authors:** Chenjing Zhang, Xiaolu Zhou, Xiaoge Geng, Yu Zhang, Jingya Wang, Yanan Wang, Jiyong Jing, Xuelong Zhou, Wensheng Pan

**Affiliations:** 1 Department of Gastroenterology, Zhejiang Provincial People’s Hospital, People’s Hospital of Hangzhou Medical College, No. 158 Shangtang Road, Hangzhou, Zhejiang China; 2 The Medical College of QingDao University, No. 308 Ningxia Road, Shinan District, Qingdao, Shandong China; 3 Zhejiang University of Technology, Hangzhou, Zhejiang China; 4 Zhejiang Provincial People’s Hospital, People’s Hospital of Hangzhou Medical College, Hangzhou, Zhejiang China; 5 Department of Anesthology, The First Affiliated Hospital of Zhejiang University, Hangzhou, Zhejiang China

**Keywords:** Cancer, Cell biology

## Abstract

Dysregulation of circular RNA (circRNA) expression is involved in the progression of cancer. Here, we aimed to study the potential function of hsa_circ_0006401 in colorectal cancer (CRC). CircRNA hsa_circ_0006401 expression levels in CRC and adjacent nontumor tissues were analyzed by real-time quantitative PCR (qRT-PCR) and circRNA in situ hybridization (RNA-ISH). Then, CRC cell proliferation was assessed by cell counting. Wound-healing and transwell assays were utilized to detect the effect of hsa_circ_0006401 on CRC migration. A circRNA-ORF construct was created, and a specific antibody against the splice junction of hsa_circ_0006401 was prepared. Finally, the proteins directly binding to hsa_circ_0006401 peptides were identified by immunoprecipitation combined with mass spectrometry. In our study, we found hsa_circ_0006401 was closely related to CRC metastasis and exhibited upregulated expression in metastatic CRC tissue samples. Proliferation and migration were inhibited in vitro when hsa_circ_0006401 expression was silenced. Downregulation of hsa_circ_0006401 expression decreased CRC proliferation and liver metastasis in vivo. A 198-aa peptide was encoded by sequences of the splice junction absent from *col6a3*. Hsa_circ_0006401 promoted CRC proliferation and migration by encoding the hsa_circ_0006401 peptide. Hsa_circ_0006401 peptides decreased the mRNA and protein level of the host gene *col6a3* by promoting *col6a3* mRNA stabilation. In conclusion, our study revealed that circRNAs generated from *col6a3* that contain an open-reading frame (ORF) encode a novel 198-aa functional peptide and hsa_circ_0006401 peptides promote stability of the host gene col6a3 mRNA to promote CRC proliferation and metastasis.

## Introduction

Globally, the morbidity and mortality of colorectal cancer (CRC) rank third and fourth among those of malignant tumors, respectively^1^. In recent years, the incidence of CRC has gradually increased, and the age of onset has become younger. Although great improvements in the diagnosis and treatment of CRC have been made, the prognosis is still not promising. Therefore, the development of new therapeutic strategies is urgently needed.

Circular RNA (circRNA) is one of the largest classes of noncoding RNA. CircRNAs are formed by a back-splicing mechanism and are abundantly present in eukaryotic transcriptomes^2^. Compared with linear noncoding RNAs, circRNA molecules have a closed circular structure that is not affected by RNA exoenzymes and is more stable^3^. Although circRNAs are usually expressed at low levels, they play important roles in regulating various physiological and pathological processes in the human body. Recent studies have found that aberrant circRNA expression is involved in tumorigenesis and progression^4–6^. For example, circRNA_102171 promotes the progression of thyroid cancer by interacting with CTNNBIP1 to activate the Wnt/β-catenin pathway^7^, high expression of circ-Foxo3 blocks the progression of the cell cycle by interacting with CDK2 through the action of promoting cell division^8^, and the circRNA cSMARCA5 promotes the expression of the tumor suppressor TIMP3 by acting as a “sponge” for miR-17-3p and miR-181b-5p, which inhibits the proliferation and migration of liver cancer cells^9^. Although an increasing number of studies suggest that circRNAs are involved in the development of tumors, circRNA research in CRC is still in its infancy. The identification of tumor-related circRNAs and the study of functional mechanisms are of great significance for the development of new diagnostic methods.

Because of the lack of the 5′ cap structure, which is considered necessary for RNA translation, circRNAs have long been considered noncoding RNAs. In recent years, studies have found that circRNAs can be used as a protein synthesis template to encode and translate proteins under certain conditions^10^. Emerging evidence has also shown that circRNA-derived proteins play important biological roles in the cell stress response, myogenesis control, and tumor progression^6,10,11^.

Here, we revealed that the expression of hsa_circ_0006401 in metastatic CRC was significantly increased compared with that in nonmetastatic CRC. Further studies revealed that silencing hsa_circ_0006401 expression with siRNA could significantly inhibit the proliferation and migration of CRC cells and promote their apoptosis and that inoculation of hsa_circ_0006401-silenced CRC cells into nude mice significantly reduced the size of subcutaneous tumors and the number of liver metastases. These results strongly suggest that hsa_circ_0006401 may play an important regulatory role in the development of CRC. In this project, we also found a novel 198-aa peptide produced from hsa_circ_0006401 that regulates the aggressive phenotype of CRC cells.

## Materials and methods

### Cell culture and transfection

The CRC cell lines SW480 and SW620 were purchased from the Shanghai Institute of Biochemistry and Cell Biology and cultured in DMEM (Gibco, Invitrogen, USA) supplemented with 10% fetal bovine serum (FBS) (Gibco, Invitrogen, USA) and 0.5% penicillin/streptomycin (Gibco, Invitrogen, USA). Cells were maintained at 37 °C in a humidified atmosphere containing 5% CO_2_. Lipofectamine^®^ 3000 reagent (Invitrogen, USA) was used for cell transfection with small interfering RNAs (siRNAs) (RiboBio, Guangzhou, China) or constructed plasmids (GenePharma, Shanghai) according to the manufacturer’s instructions. The siRNA sequences were as follows:

siRNA#1 5′ ACAGAAAUGUUCCGAAUAA dTdT 3′,

siRNA#2 5′ CUCUCACUGAAACAGAAAU dTdT 3′.

### Patient samples

This work was approved by the Research Ethics Committee of Zhejiang Provincial People’s Hospital, Hangzhou Medical College (code: 2020QT084). Twelve samples from CRC patients were collected from Zhejiang Provincial People’s Hospital between May 2017 and July 2020. All the enrolled patients in this study had never received preoperative therapy. All tissues were histologically diagnosed as CRC. Patient information is shown in Table 1. Tissue samples were collected and frozen in liquid nitrogen immediately after surgical resection. The sample size was estimated according to our preliminary experimental data.

**Table 1:** Clinical characteristics of the patients.

Characteristics	qRT-PCR	FISH	IHC
Total number	12	10	12
Gender (male/female)	8/4	6/4	8/4
Age (year, mean)	58.3 ± 4.2	61.1 ± 8.5	58.3 ± 4.2
Adenocarcinoma (yes/no)	12/0	10/0	12/0
*Pathology stage*			
pTa-pT1	0	0	0
pT2-T4	12	10	12
*Histologic differentiation*			
Well differentiated	1	1	1
Moderately differentiated	11	9	11
Poorly differentiated	0	0	0
Lymphatic metastasis (yes/no)	6/6	6/4	6/6
Vascular invasion (yes/no)	4/8	2/8	4/8

### RNA extraction and purification

Total RNA was extracted from tissue and cell samples with TRIzol (Invitrogen, USA), and the RNA concentration and purity were checked by the Agilent 2100 Bioanalyzer according to the manufacturer’s protocol (Agilent Technologies, USA). RNA was then reverse transcribed into cDNA using the SuperScript^TM^ IV First-Strand Synthesis System (Invitrogen, USA) according to the instructions provided by the manufacturer.

### qRT-PCR and mRNA decay analyses

Cells transfected with indicated siRNAs were directly harvested or treated with 1 μg/ml Actinomycin D (mRNA decay, Sigma-Aldrich) and harvested at indicated time points. Hsa_circRNA_0006401 and mRNA level was detected by qRT-PCR utilizing TB Green^TM^ Premix DimerEraser^TM^ (Perfect Real Time) on the ABI 7900 Real-Time PCR System (Thermo Fisher Scientific, USA). The reaction conditions were set according to the manufacturer’s protocol (Takara, Beijing). The human GAPDH reference gene was used as an internal control. For mRNA decay assay, 28S RNA was used as an internal control. All assays were conducted in triplicate. The PCR products were sent to GENESEED (Guangzhou, China) for sequencing to ensure the accuracy of circRNA detection. Primer information is as follows:

hsa_circ_0006401 Forward, 5ʹ-TGGCTCTCACTGAAACAGAAATG-3ʹ;

Reverse, 5ʹ-GTCGTCAC TGGGTTGGATGTAG-3ʹ

TGFβ1 Forward, 5ʹ-CGGCTGC TGCTGAAAGCCGACCA-3ʹ;

Reverse, 5ʹ-GGTCGGGGCCAAAAGCGTGT-3ʹ

COL6A3 Forward, 5ʹ-ATGAGGAAACATCGGCACTTG-3ʹ;

Reverse, 5ʹ-GGGCATGAGTTGTAGGAAAGC-3ʹ

GAPDH Forward, 5ʹ-AGTCAGCATT TCACAAGACCTC-3ʹ;

Reverse, 5ʹ-CAGGCGAAGATGTTCTGGC-3ʹ;

28S RNA Forward, 5ʹ-CCCAGTGCTCTGAATGTCAA-3ʹ;

Reverse, 5ʹ-AGTGGGAATCTCGTTCATCC-3ʹ.

### CircRNA in situ hybridization (RNA-ISH)

Ribo^TM^ Fluorescent in Situ Hybridization Kit and RiboTM hsa_circ_0006401 FISH Probe Mix were purchased from Ribobio company. CRC tumor and para tumor tissue slides were deparaffinized, rehydrated, and then disposed of according to the instructions provided by the manufacturer (Ribobio, RN: R11060.4). Finally, the slides were evaluated with a fluorescence microscope.

### Flow cytometry

Adherent CRC sw480 cells were harvested and washed with PBS supplemented with 0.5% bovine serum albumin (BSA). After fixation with 70% ethanol at −20 °C overnight, the sw480 cells were resuspended in PBS supplemented with 40 μg/ml PI at 37 °C for 30 min, and 100 μg/ml RNase A was subsequently added to the cells and incubated in a 4 °C dark room for 30 min. Cell apoptosis was determined with a flow cytometer (BD Biosciences). All assays were conducted in triplicate.

### Xenograft assay

The studies received approval from the medical ethical committee of The Zhejiang Provincial People’s Hospital. SW620 cells (2 × 10^6^) in 150 μl of PBS were randomly subcutaneously injected into 4-week-old male BALB/c nude mice. Two weeks after cancer cell inoculation, the tumors were isolated, and tumor volume was determined using the following formula: 0.5236 × *L*1 × (*L*2)2, *L*1 is the long axis and *L*2 is the short axis of the tumor. The tumor tissue and liver of nude mice were collected and fixed in a 10% buffered formaldehyde solution, and hematoxylin and eosin (H&E) staining was applied to assess tumor invasion and metastasis independently by two physicians. The physicians were blind to these slides. The sample size was estimated according to published articles.

### Hematoxylin and eosin staining

Tissue sections were formalin fixed, paraffin embedded, and stained with hematoxylin and eosin (H&E). Images of tumors were acquired with a light microscope. Ten sections were randomly chosen to analyze local invasion and liver metastasis. The liver lesion number was quantified by ImageJ software (version 2.1.4).

### Immunohistochemistry

Fresh CRC and paratumor tissues were washed with PBS and processed into tissue blocks. Then, the tissue blocks were fixed, embedded, sectioned at a thickness of 5 µm, attached to a polylysine slide overnight at 60 °C and dewaxed. An antigen retrieval solution and a blocking solution were added to the sections. Then, primary antibodies (HuaAn Biotechnology Co., Ltd, HAPM0617) were applied to the sections and incubated at 4 °C overnight. Next, the sections were incubated with a biotinylated secondary antibody for 20 min at room temperature. The staining intensity of the hsa_circ_0006401 peptide was scored independently by two physicians.

### Western Blot

Proteins were isolated from CRC cells and incubated with primary antibody detecting hsa_circ_0006401 peptide (1:1000 dilution, HuaAn Biotechnology Co., Ltd), Col6a3 (1:1000 dilution, sinobiological, 16125-T48), HA-probe (Santa Cruse, sc-57592), and GAPDH (1:1000 dilution, Abcam, ab181602) was used as a control. Amino acid sequence for hsa_circ_0006401 peptide antibodies were as follows: 1. HAPL0559 147-161aa CSFSTKKSQPPPPQPA; 2. HAPM0617 splice junction TEMFRITLLQVLHPTQC. The anti-rabbit secondary antibody was then applied (1:1000 dilution, Abcam, ab6721). Finally, enhanced chemiluminescence was utilized to observe immunoreactive proteins.

### Co-immunoprecipitation

SW480 cells were washed with PBS and lysed by cold lysis buffer (1% TritonX-100, 50 mM Tris-7.5, 1 mM EDTA, 150 mM NaCl, and protease inhibitors). The supernatant was collected and incubated with antibody HAPM0617. Then, the protein G beads were incubated with the lysates. The beads were washed by cold lysis buffer, and the protein loading buffer was added to the beads, then were forwarded to Hangzhou Molecular Diagnostic Bio-tech Company. Ltd for Mass spectrometry analysis.

### Statistical analysis

Statistical analysis was carried out by using SPSS 21.0 software (SPSS, USA). Differences between individual groups were compared by Student’s *t*-test. All data are presented as the means ± standard deviation (SD). A *P*-value < 0.05 was considered significant. Data from the experiments are expressed as the means ± SD from at least three independent experiments.

## Results

### Hsa_circ_0006401 expression was upregulated in metastatic CRC tissue

**Fig. 1:**
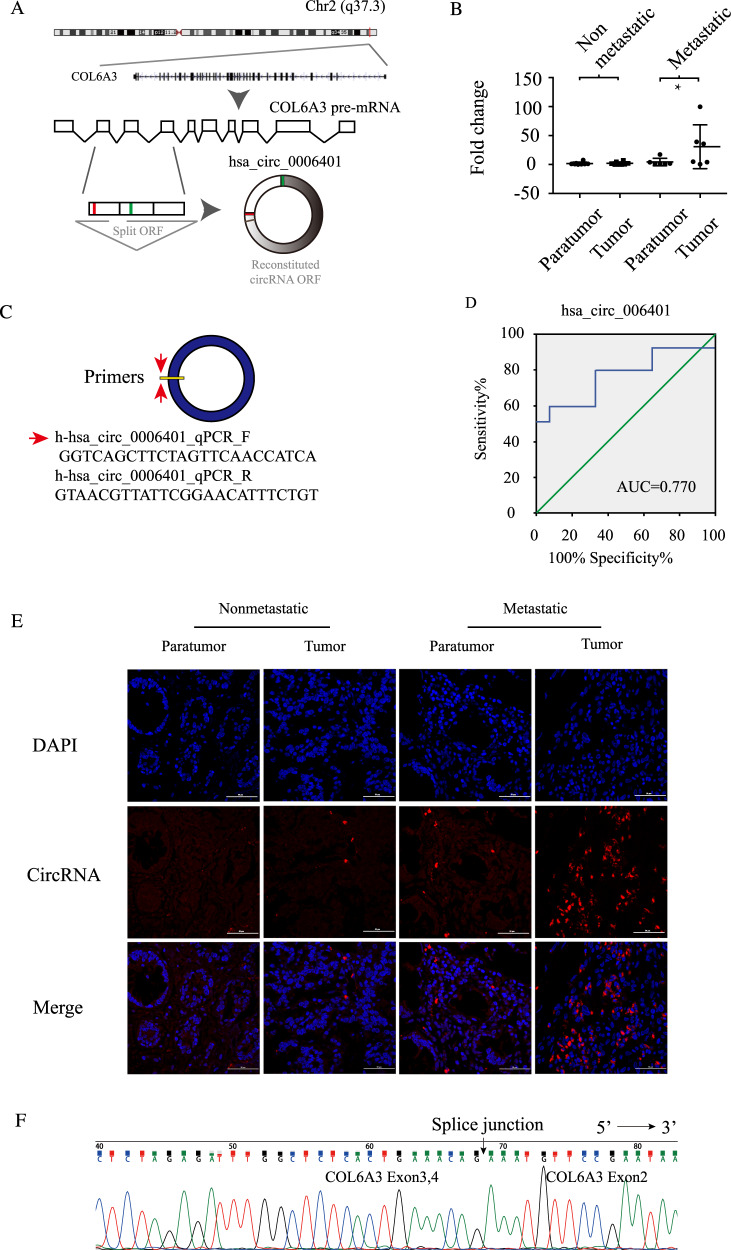
Analysis of the expression level of hsa_circ_0006401 in CRC and corresponding normal tissue specimens. **A** Schematic representation of the back splicing of hsa_circ_0006401. **B** Gene expression level of hsa_circ_0006401 in CRC patients in either the metastasis or control group. **C** Primers for hsa_circ_0006401. **D** ROC curve analysis of hsa_circ_0006401 in CRC patients. **E** Reprehensive images of hsa_circ_0006401 in metastatic and nonmetastatic CRC tissue samples. **F** Sequencing of PCR products with a splice junction. Error bars indicate SD, *P < 0.05.

Hsa_circ_0006401 is back-spliced from three exons (exons 2, 3, and 4) of *col6a3* on Chr2(q37.3) (Fig. 1A). To investigate the potential function of hsa_circ_0006401 in CRC, the expression level of hsa_circ_0006401 was analyzed by qRT-PCR and compared between 12 pairs of CRC tissue and corresponding paratumor tissue specimens. We found that the hsa_circ_0006401 expression level was higher in most of the CRC tissue samples and significantly related to lymph node metastasis (Table 2). Moreover, in regard to the expression of hsa_circ_0006401 in metastatic CRC, qRT-PCR was performed to compare metastatic and nonmetastatic CRC tissue samples. As shown in Fig. 1B, hsa_circRNA_0006401 expression was upregulated in CRC patients with lymphatic metastasis. Primers are shown in Fig. 1C. To evaluate the diagnostic potential of hsa_circ_0006401 for CRC, receiver operating characteristic (ROC) curve analysis was carried out, and we found that the area under the curve (AUC) of the selected circRNAs was 0.770 (95% CI 0.549–0.991, *P* = 0.041) (Fig. 1D). An RNA-ISH assay was also applied to assess the hsa_circ_0006401 expression level. We found that hsa_circ_0006401 was barely expressed in normal colorectal tissue specimens and was expressed at higher levels in metastatic CRC tissue samples than in nonmetastatic CRC tissue samples (Fig. 1E). Moreover, to further validate our results, the sequences of the PCR products were identified and confirmed to be the indicated circRNAs with a back-spliced junction (Fig. 1F).

**Table 2:** Correlation between hsa_circ_0006401 expression and clinical features in CRC.

Characteristics	No. (%)	Has_circ_0006401 expression		*p*-value
		Low(%)	High(%)	
*Gender*				
Male	8 (67)	4 (67)	4 (67)	0.264
Female	4 (33)	2 (33)	2 (33)	
*Age*				
<60	7 (58)	4 (67)	3 (40)	0.149
≥60	5 (42)	2 (33)	3 (60)	
*Tumor size*				
<5 cm	2 (50)	3 (50)	3 (50)	0.319
≥5 cm	10 (50)	3 (50)	3 (50)	
*Pathology stage*				
pTa-pT1	0 (0)	0 (0)	0 (0)	0.899
pT2-T4	12 (100)	6 (100)	6 (100)	
Grade				
Low	11 (92)	6 (100)	5 (83)	0.781
High	1 (8)	0 (0)	1 (17)	
Lymphatic metastasis				
Yes	6 (50)	1 (17)	5 (83)	0.040*
No	6 (50)	5 (83)	1 (17)	
Vascular invasion				
Yes	4 (33)	2 (33)	2 (33)	0.548
No	8 (67)	4 (67)	4 (67)	
Total	12	6	6	

Chi-square test. *p < 0.05.

### Hsa_circ_0006401 promotes an aggressive phenotype in CRC cells in vitro

**Fig. 2:**
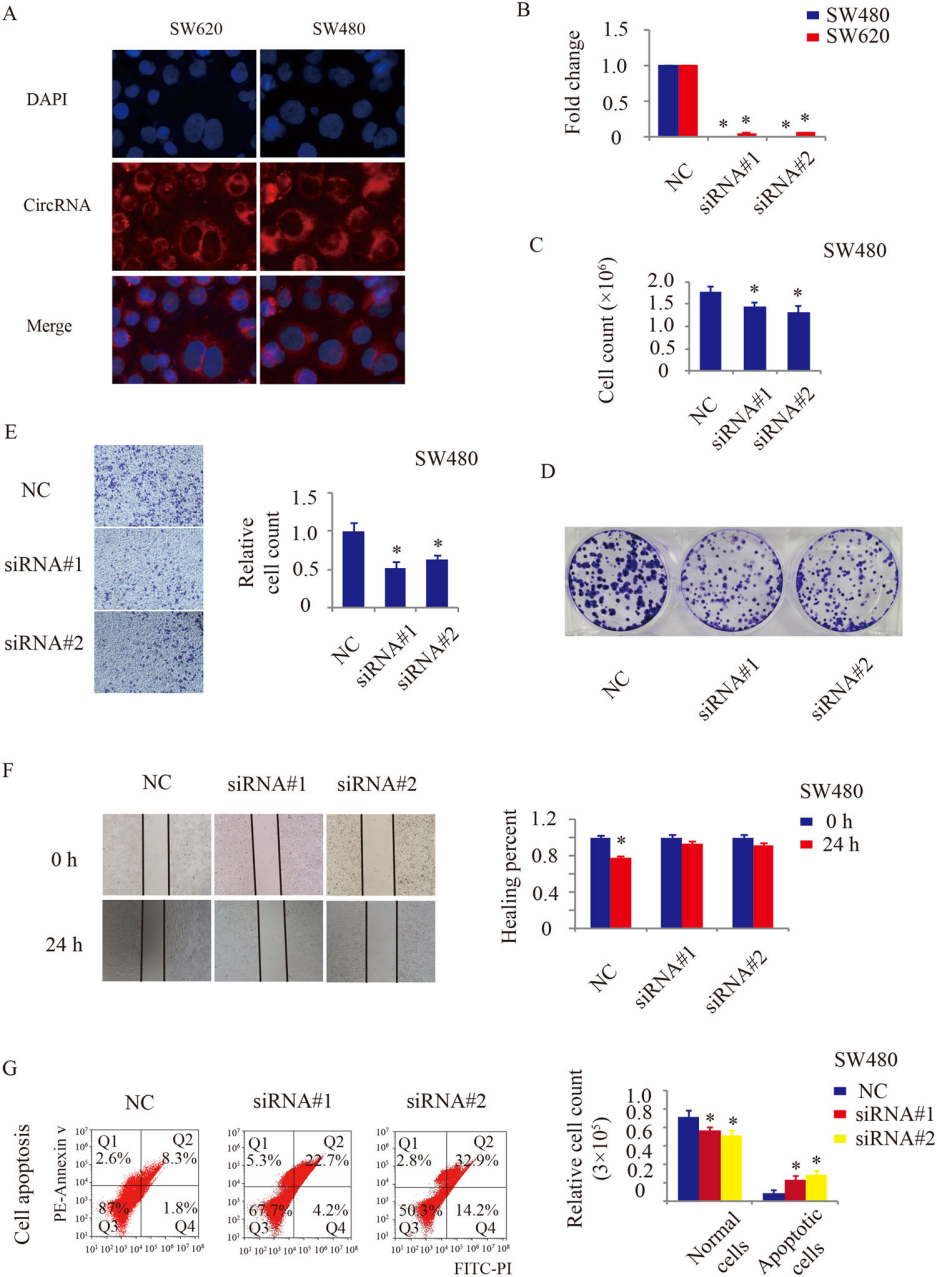
Hsa_circ_0006401 promoted the proliferation and migration of CRC cells in vitro. **A** Representative images of the expression and location of hsa_circ_0006401 in SW480 and SW620 cells. **B** Silencing efficiency of two siRNAs in the CRC cell lines SW480 and SW620 by qRT-PCR. **C** Cell counts of CRC cells in the control group and hsa_circ_0006401 downregulation group. **D** Clone forming assay to detect the proliferative ability of CRC cells in the control group and hsa_circ_0006401 downregulation group. **E** Transwell assay to detect the migratory ability of CRC cells in the control group and hsa_circ_0006401 downregulation group. **F** Wound-healing assay to detect the migratory ability of CRC cells in the control group and hsa_circ_0006401 downregulation group. **G** Flow cytometry to assess cell apoptosis in the control group and hsa_circ_0006401 downregulation group. Error bars indicate SD, *p < 0.05, **p < 0.01, ***p < 0.001.

To further confirm the function of hsa_circRNA_0006401, the location and expression of hsa_circRNA_0006401 were explored by circRNA in situ hybridization. As shown in Fig. 2A, hsa_circRNA_0006401 mainly existed in the cytoplasm of SW480 and SW620 cells. Next, two siRNAs were designed according to the splice junction of hsa_circRNA_0006401. As shown in Fig. 2B, hsa_circRNA_0006401 was perfectly downregulated in SW480 and SW620 cells by both siRNAs (Fig. 2B). Then, cell proliferation was analyzed, and we found that compared to negative control treatment, downregulation of hsa_circRNA_0006401 by both siRNAs decreased CRC cell counts (Fig. 2C) and clone forming numbers (Fig. 2D). Moreover, wound-healing and transwell assays were utilized to investigate CRC cell migration. As shown in Fig. 2E, F, the numbers of migrated CRC cells were significantly decreased by silencing hsa_circRNA_0006401. Finally, cell apoptosis was also evaluated by flow cytometry, and we found that hsa_circRNA_0006401 knockdown increased apoptotic CRC cell numbers (Fig. 2G). Overall, hsa_circRNA_0006401 promotes an aggressive phenotype in CRC cells in vitro.

### Hsa_circ_0006401 increased the proliferation and metastasis of CRC in vivo

**Fig. 3:**
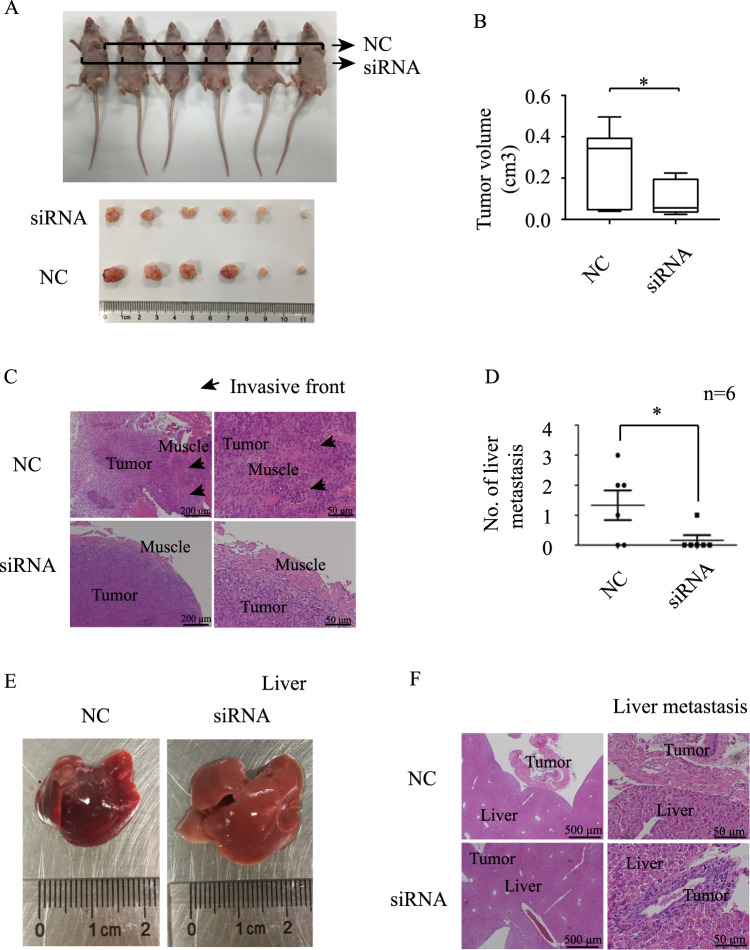
Hsa_circ_0006401 promoted the proliferation and metastasis of CRC in vivo. **A** Cells were subcutaneously injected into nude mice. All injected mice formed tumors. Tumor were isolated, and tumor volume was measured. **B** Box plot of tumor volume in nude mice. *n* = 6. *p < 0.05. **C** Tumors and the surrounding tissues from mice in the control and hsa_circ_0006401-silenced groups were fixed and subjected to hematoxylin and eosin staining. **D** The number of liver metastases and the metastatic burden was determined and graphed. *p < 0.05. **E** Representative images of the livers of mice from different groups are shown. **F** Representative images of hematoxylin and eosin staining of the livers of mice from different groups are shown. Error bars indicate SD.

To evaluate the function of hsa_circRNA_0006401 in vivo, control SW620 CRC cells and hsa_circRNA_0006401-silenced SW620 CRC cells were subcutaneously injected into the right and left subaxillary regions, respectively, of nude mice. Two weeks after cancer cell inoculation, the mice were sacrificed, and the tumors were isolated. As shown in Fig. 3A, B, the tumor size of the hsa_circRNA_0006401-silenced tumors was much smaller than that of the control tumors. As a negative control group, PBS-injected mice did not form tumors. Tumors in situ and the surrounding tissues as well as the liver were fixed and subjected to H&E staining. The results showed that the deep tumor margins of control group were distinct and invaded nearby structures, such as skeletal muscles. However, the margins in the hsa_circRNA_0006401-silenced CRC cell-formed tumors were well encapsulated with a noninvasive nature (Fig. 3C). The numbers of liver metastases were also assessed, and we found that hsa_circRNA_0006401 knockdown significantly decreased the number of liver metastasis lesions in nude mice (Fig. 3D, E). H&E staining results for nude mouse livers are shown in Fig. 3F.

### Hsa_circ_0006401 encodes a small peptide

**Fig. 4:**
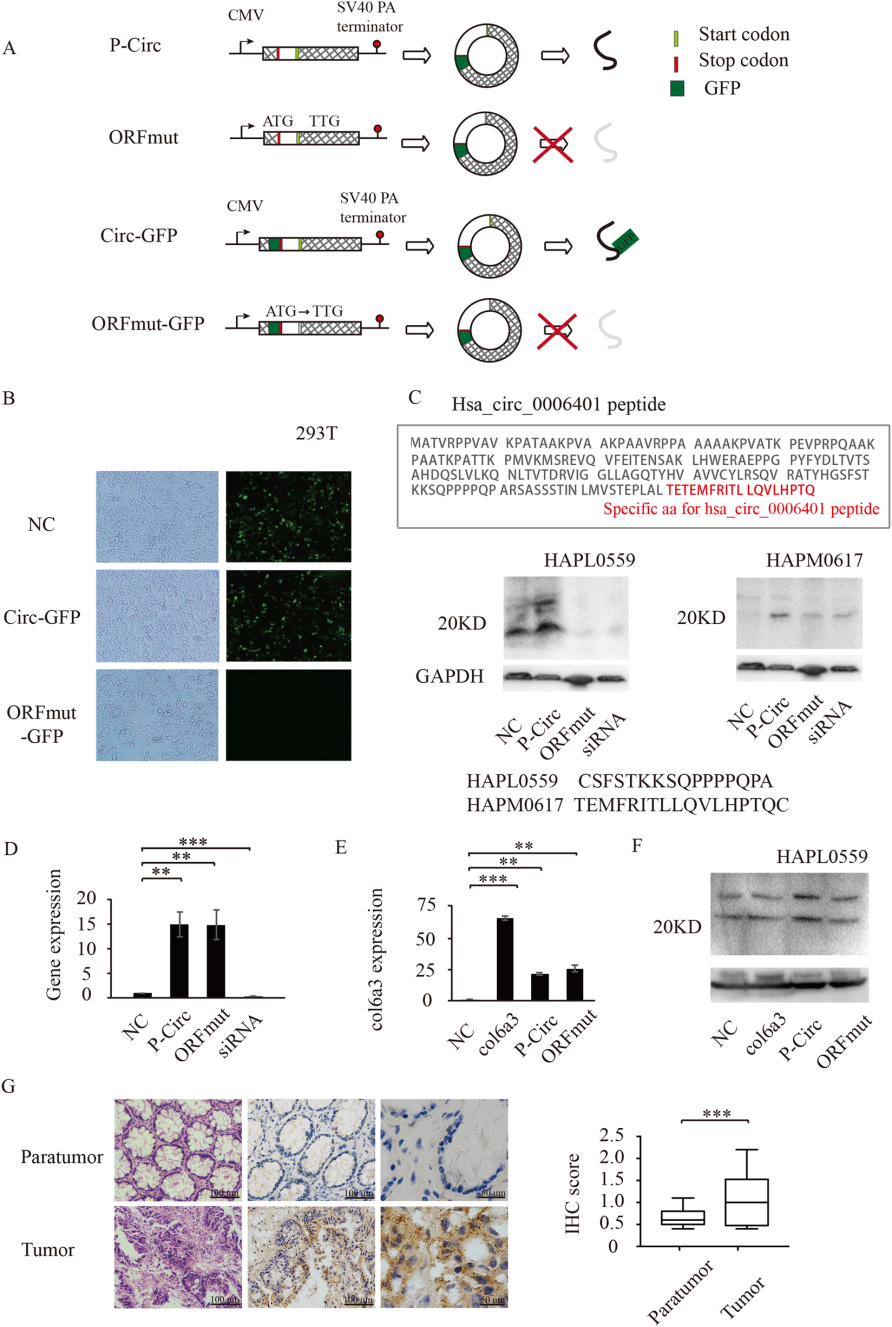
Hsa_circ_0006401 encoded a novel peptide. **A** Schematic representation of plasmid construction. **B** Detection of GFP fluorescence. The indicated constructs were transfected into HeLa cells for 24 h. **C** Western blot analysis with anti-hsa_circ_0006401 antibodies to evaluate proteins from SW620 cells transfected with different constructs. NC (negative control), p-Circ (hsa_circ_0006401 circRNA level was highly expressed), ORFmut (ATG start codon of p-Circ was mutated to TTG), siRNA (hsa_circ_0006401 circRNA level was silenced by siRNA). GAPDH was used as a loading control. **D** qRT-PCR analysis of hsa_circ_0006401 cirRNA expression level of different groups. **E** qRT-PCR analysis of hsa_circ_0006401 cirRNA expression level of different groups of SW480 cells. NC (negative control), col6a3 (col6a3 overexpression), p-Circ (hsa_circ_0006401 circRNA level was highly expressed), ORFmut (ATG start codon of p-Circ was mutated to TTG). **F** Western blot analysis with anti-hsa_circ_0006401 antibodies (HAPL0559) to evaluate proteins from SW480 cells transfected with different constructs. NC (negative control), col6a3 (col6a3 overexpression), p-Circ (hsa_circ_0006401 circRNA level was highly expressed), ORFmut (ATG start codon of p-Circ was mutated to TTG). **G** Reprehensive images of IHC analysis with an antibody (HAPM0617) to evaluate proteins from colon cancer tissues and normal colon tissues (left panel). IHC scores were calculated (right panel). Error bars indicate SD, *p < 0.05, **p < 0.01, ***p < 0.001.

Hsa_circ_0006401 originates from the circularization of the second to fourth exons of the host gene *col6a3*. A 594-nt open reading frame (ORF) is present in hsa_circ_0006401, spanning across the splice junction, which has the potential to encode a 198-aa peptide (Fig. 4A). To determine whether this ORF has peptide-encoding potential, an expression vector with a circular transcript-producing capacity was adopted. P-Circ contained the second to fourth exons of the *col6a3* gene and was able to express hsa_circ_0006401 circRNA at high levels. The sequence produced from this construct was perfectly consistent with that of hsa_circ_0006401 (data not shown). A construct was derived from p-Circ-GFP that contained a GFP sequence without an AUG initiation codon immediately upstream of the STOP codon, such that a GFP fusion protein could be produced when a circular template formed. We observed GFP expression in P-Circ-GFP-transfected 293T cells. However, a construct with a mutation in the ORF start codon (the start codon ATG was mutated to TTG) did not exhibit GFP fusion protein expression after transfection (Fig. 4B).

To confirm the peptide encoded by the hsa_circ_0006401 ORF in CRC, we produced two antibodies (HAPL0559, HAPM0617) against the hsa_circ_0006401 peptides, one of which was against unique reads of the circular junction (HAPM0617). As shown in Fig. 4C, the hsa_circ_0006401 peptides were highly expressed in the P-Circ-transfected CRC group compared to the control group. However, there was barely any hsa_circ_0006401 peptide expression when the hsa_circ_0006401 ORF start codon was mutated or hsa_circ_0006401 circRNA expression was silenced. Moreover, hsa_circ_0006401 circRNA expression level were also detected by qRT-PCR, and we found hsa_circ_0006401 circRNA was highly expressed in both P-Circ and ORFmut transfected CRC cells (Fig. 4D). Taken together, these data indicate that the hsa_circ_0006401 peptides are encoded by circRNA hsa_circ_0006401 in CRC. To further exclude that the hsa_circ_0006401 peptide is produced by its linear mRNA, col6a3 overexpression plasmid and control vector were transfected to CRC cells. As shown in our results, when col6a3 mRNA level was significantly increased (Fig. 4E) hsa_circ_0006401 peptides expression didn’t obviously altered (Fig. 4F), suggesting hsa_circ_0006401 peptide was not derived from linear mRNA. Moreover, IHC staining was performed to assess hsa_circ_0006401 peptide expression in CRC and paratumor tissue samples from twelve CRC patients. We found that the IHC scores for the hsa_circ_0006401 peptides were higher in the CRC tissue samples than in the paratumor tissue specimens and closely related to lymphatic metastasis (Fig. 4G).

### Hsa_circ_0006401 peptide promotes CRC Growth and Migration

**Fig. 5:**
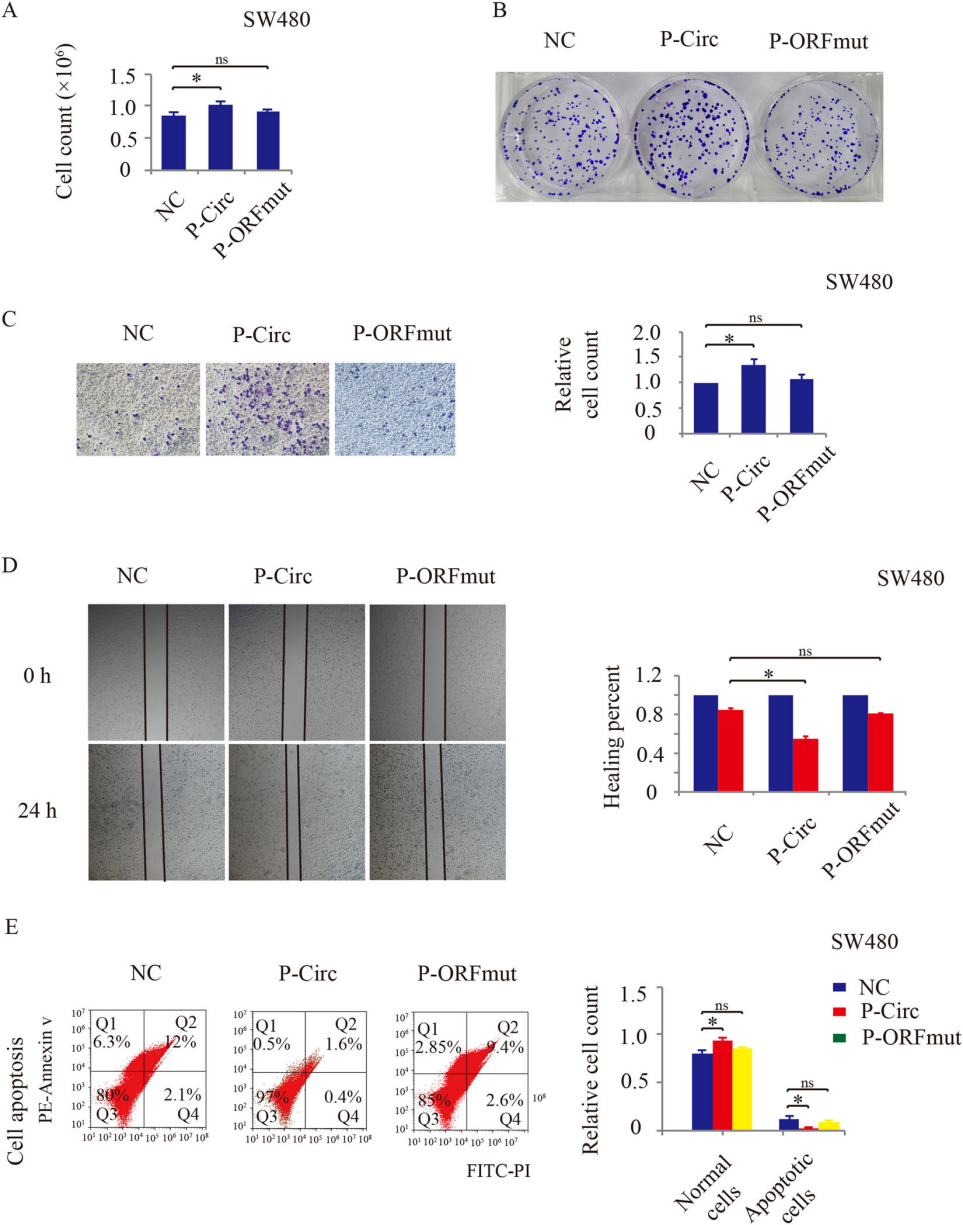
Function of the ORF in CRC. **A** Cell counts of SW480 CRC cells transfected with different constructs. **B** Clone forming assay to detect the proliferative ability of SW480 CRC cells transfected with different constructs. **C** Transwell assay to detect the migratory ability of SW480 CRC cells transfected with different constructs. **D** Wound-healing assay to detect the migratory ability of SW480 CRC cells transfected with different constructs. **E** Flow cytometry assay to assess cell apoptosis in SW480 CRC cells transfected with different constructs. NC (negative control), p-Circ (hsa_circ_0006401 circRNA level was highly expressed), ORFmut (ATG start codon of p-Circ was mutated to TTG). Error bars indicate SD, *p < 0.05, ns represents no significance.

To investigate whether hsa_circ_0006401 regulates CRC growth, migration and metastasis by encoding the hsa_circ_0006401 peptides, the p-Circ and ORFmut constructs were transfected into CRC cells. Then, clone formation assay, transwell assay and wound-healing assay were applied to assess the function of hsa_circ_0006401 peptides on ability of proliferation and migration of CRC cells. As shown in Fig. 5, transfection of p-Circ, which increased both the hsa_circ_0006401 circRNA (Fig. 4E) and peptide expression level (Fig. 4F), enhanced CRC growth (Fig. 5A), clone formation (Fig. 5B) and migration (Fig. 5C, D), but decreased CRC apoptosis (Fig. 5E). In contrast, transfection of ORFmut (contained a mutated start codon for the hsa_circ_0006401 ORF) which merely alter circRNA hsa_circ_0006401 expression (Fig. 4E) but not the hsa_circ_0006401 peptide expression level (Fig. 4F), did not alter CRC growth (Fig. 5A), clone formation (Fig. 5B), migration (Fig. 5C, D) or apoptosis (Fig. 5E). Together, the results indicate that hsa_circ_0006401 promotes an aggressive phenotype in CRC cells by encoding the hsa_circ_0006401 peptides.

### Hsa_circ_0006401 peptides regulate COL6A3 mRNA expression

**Fig. 6:**
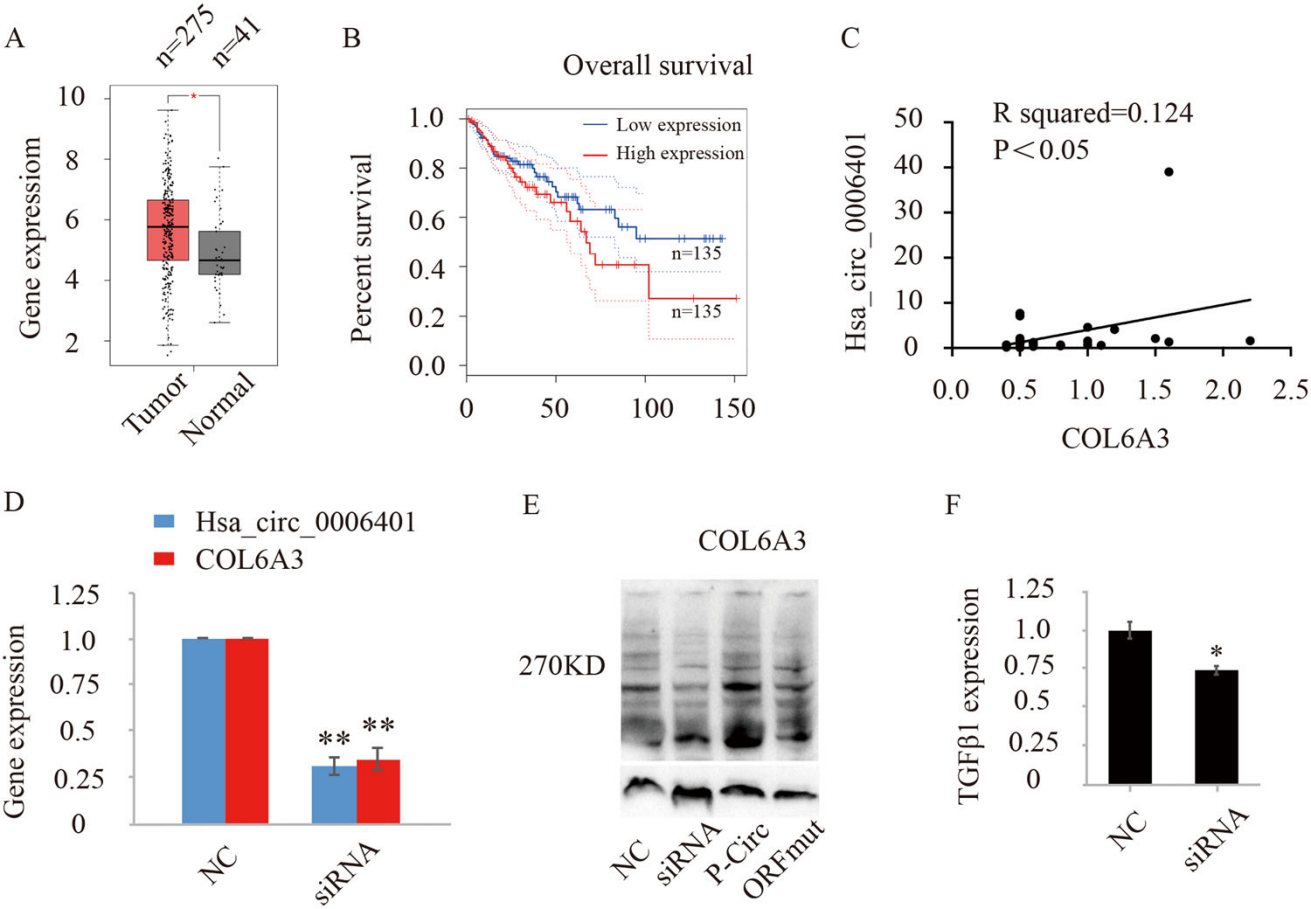
Hsa_circ_0006401 peptides regulate COL6A3 mRNA expression. **A** Gene set analysis of COL6A3 expression in normal tissue and colon cancer using TCGA cancer browser. The number of patients from each subtype is indicated below the box plot. **B** COL6A3 expression was assessed by Kaplan–Meier survival analysis for 5-year overall survival outcome in 270 colon cancer patients. **C** The correlation of hsa_circ_0006401 and COL6A3 mRNA in 12 human CRC tissues. *p < 0.05. **D** COL6A3 mRNA expression level in negative control SW620 cancer cells (NC) and hsa_circ_0006401 silencing SW620 cancer cells (siRNA). **p < 0.01. **E** COL6A3 protein expression level in SW620 cells transfected with different constructs. NC (negative control), p-Circ (hsa_circ_0006401 circRNA level was highly expressed), ORFmut (ATG start codon of p-Circ was mutated to TTG), siRNA (hsa_circ_0006401 circRNA level was silenced by siRNA). **F** TGFβ1 mRNA expression level in negative control SW620 cancer cells (NC) and hsa_circ_0006401 silencing SW620 cancer cells (siRNA). *p < 0.05. Error bars indicate SD.

The collagen alpha-3 (VI) chain (COL6A3) is a key component of the extracellular matrix and highly expressed in multiple malignants^12^. Endotrophin, the cleaved C5 domain fragment of COL6A3, can directly regulate the malignancy of cancer cells via TGFβ-dependent mechanisms^12^. Data from TCGA showed that compared with normal tissues, COL6A3 expression level is higher in colon cancer tissues (Fig. 6A). Moreover, high COL6A3 gene expression level predicts unfavorable prognosis (Fig. 6B). To further study whether the hsa_circ_0006401 peptide contributes to modulating or controlling the activity of COL6A3. We found that the expression of hsa_circ_0006401 was positively correlated with linear COL6A3 mRNA in human tissues (Fig. 6C). Silencing hsa_circ_0006401 circRNA level decreased the gene expression level of the host gene *col6a3* (Fig. 6D). Moreover, COL6A3 expression was also downregulated by silence of hsa_circ_0006401 circRNA level. In p-Circ transfected cells, which encoded high level of hsa_circ_0006401 peptides, mutation of start codon of hsa_circ_0006401 ORF inhibted expression level of COL6A3 (Fig. 6E). Down-regulation of hsa_circ_0006401 also decreased TGFβ1 gene expression (Fig. 6F).

Taken together, the results indicated linear RNA COL6A3 expression level was regulated by hsa_circ_0006401 peptides expression.

### Hsa_circ_0006401 peptides promote the stability of col6a3 mRNA

**Fig. 7:**
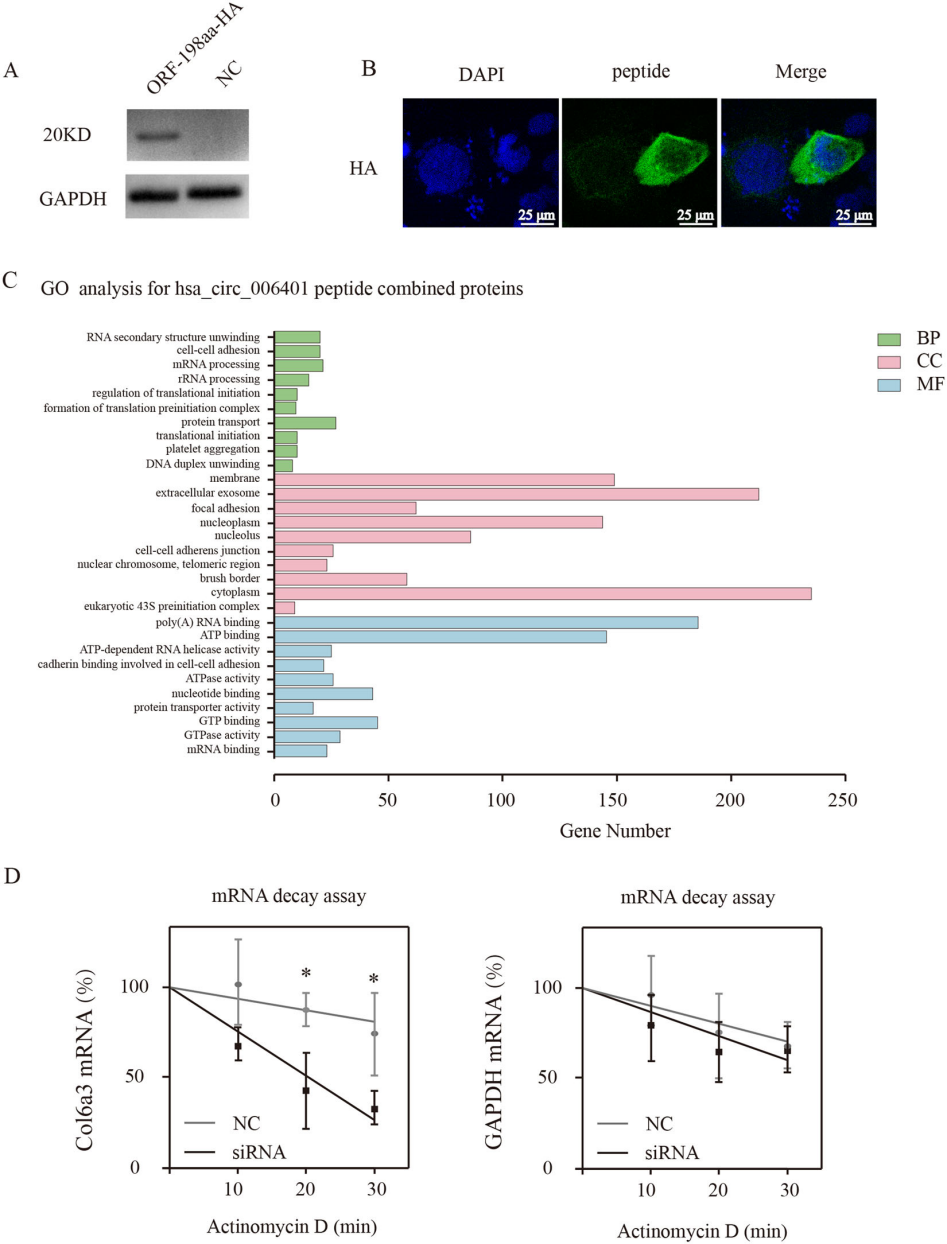
Hsa_circ_0006401 peptides promote COL6A3 mRNA stabilization. **A** HA tag expression in negative control SW480 cancer cells (NC) and hsa_circ_0006401 ORF-198aa-HA tag plasmid transfected SW480 cancer cells (ORF-198aa-HA). **B** Representative images of the location of hsa_circ_0006401 peptide in SW480 cells. **C** Gene ontology (GO) analysis for hsa_circ_0006401 peptide combined proteins. BP represents biological processes, CC represents cellular components and MF represents molecular functions. **D** Decay of col6a3 and GAPDH mRNA was monitored in SW480 cells treated with Actinomycin D with indicated time points. NC (negative control), siRNA (hsa_circ_0006401 circRNA level was silenced by siRNA). Min represents minutes. Error bars indicate SD, and data were fitted by linear regression. *p < 0.05.

To further study the molecular activity of the peptide derived from hsa_circ_0006401, we inserted the full-length sequence of hsa_circ_0006401 ORF (ORF-198aa) into a plasmid with HA tag (Fig. 7A). The immunostaining results displayed that this protein was mainly located in the cytoplasm of CRC cells (Fig. 7B), indicating hsa_circ_0006401 peptides might post-transcriptionally regulate col6a3 mRNA level. Next, the proteins directly binding to hsa_circ_0006401 peptides were identified by immunoprecipitation combined with mass spectrometry. Gene ontology (GO) analysis was also performed to annotate the function of the hsa_circ_0006401 peptide binding proteins (Fig. 7C). Notably, we found these proteins were strongly linked to poly(A) RNA binding and mRNA processing (Fig. 7C). Since the decrease of linear mRNA level at steady state could result from altered transcription or mRNA stability, col6a3 mRNA degradation was further analyzed by mRNA decay assay. We found the knockdown of hsa_circ_0006401 peptides led to an approximately two-fold accelerated decay of the *col6a3* mRNA, but did not affect the decay of GAPDH mRNA (Fig. 7D), suggesting that hsa_circ_0006401 peptides are essential and promote the stability of linear mRNA COL6A3. Taken together, the results indicated hsa_circ_0006401 peptides promote the stability of *col6a3* mRNA.

## Discussion

circRNA is a novel type of endogenous RNA that is widely and stably present in eukaryotic cells. Due to the lack of a cap structure and polyA tail, circRNAs form a covalently closed loop, are highly resistant to RNase activity and are conserved across species. With the rapid development of RNA deep-sequencing technology, a few cancer-related circRNAs have been identified. However, the biological functions and mechanisms of most circRNAs are largely unexplored. Accumulating studies also suggest that circRNAs participate in the tumorigenesis of human cancer and hold promise to become new diagnostic and prognostic biomarkers for various cancers.

Hsa_circ_0006401 is derived from its host gene *col6a3* and has unknown molecular structures in CRC. In this study, we found that the expression of hsa_circ_0006401 was closely related to lymph node metastasis. Further investigation suggested that compared to CRC tissue samples from nonmetastatic patients, cancer tissue samples from metastatic patients showed hsa_circ_0006401 upregulation. In vitro and in vivo studies showed that hsa_circ_0006401 promoted CRC growth, migration, and metastasis and inhibited CRC apoptosis. Together, these results indicate that hsa_circ_0006401 may have a potential function in the tumorigenesis of CRC. The potential diagnostic capacity of hsa_circ_0006401 was also evaluated in our study, and we revealed that hsa_circ_0006401 might serve as a novel biomarker for metastatic CRC patients. To the best of our knowledge, this is the first report to reveal the functional and diagnostic value of hsa_circ_0006401 in CRC. Our results indicate that hsa_circ_0006401 may serve as a potential biomarker of CRC and is involved in the regulation of CRC tumorigenesis, which provide the new insight that the circularization of the three exons spliced from the pre-mRNA col6a3 may maintain functions consistent with those of the host gene.

The mechanisms involved in the regulatory function of circRNAs are more complex. Some circRNAs may be sponges for microRNAs (miRNAs)^13^ or interact with RNA-binding proteins (RBPs)^14^ to moderate gene expression. Recently, several studies have suggested that circRNAs can be translated and encode functional proteins in a cap-independent manner through the internal ribosomal entry site (IRES)^15,16^. Nevertheless, genome-wide studies have demonstrated that translation of circRNAs, which is driven by short sequences containing N6-methyladenosine (m6A) as the IRES, is widespread in human cells^17^. Emerging evidence also suggests that circRNA-derived proteins play important biological roles in cellular responses to environmental stress, myoblast proliferation and tumorigenesis.

Hsa_circ_0006401 is produced by 2–4 exons of its host gene *col6a3* and is localized in the cytoplasm. In our study, we found ORFs across the back-spliced junction of hsa_circ_0006401. To study whether hsa_circ_0006401 has protein-coding potential, a construct producing a high hsa_circ_0006401 circRNA expression level was generated, and a GFP sequence without an AUG initiation codon was inserted immediately upstream of the ORF termination codon. The GFP-fusion protein was detected by fluorescence microscopy in 293T cells. However, most GFP expression was blocked when the AUG codon of the hsa_circ_0006401 ORF was mutated. Western blotting results were consistent with the immunostaining results. With comparison to the host gene, we found that a novel 198-aa peptide with an additional amino acid might be produced from this ORF, which was absent in the host gene *col6a3* mRNA transcript.

To further confirm peptide expression, the peptide sequence spanning the back–splice junction, unambiguously identified as hsa_circ_0006401-encoded products, was detected by IHC. The peptide was found to be expressed in both the nucleus and cytoplasm in human colon cancer and paratumor tissue specimens. Therefore, the hsa_circ_0006401 peptide was considered to be naturally endogenously produced in human colon cancer tissues. Hsa_circ_0006401 peptide expression was also confirmed in SW480 colon cancer cells. Moreover, mutation of the hsa_circ_0006401 ORF prevented the increased proliferation and migration of CRC cells induced by overexpression of hsa_circ_0006401, suggesting that hsa_circ_0006401 may promote an aggressive phenotype in CRC cells by encoding the hsa_circ_0006401 peptide. Recently, a few studies also reported that circRNAs might encode functional peptides or proteins. For example, the circRNA Circ-ZNF609 is translated into a protein and regulates myogenesis. Furthermore, the circRNA-derived protein SHPRH-146aa suppresses glioma tumorigenesis by protecting full-length SHPRH from degradation by the ubiquitin–proteasome. Taken together, these results emphasize the potentially important roles of peptides encoded by circRNAs.

To date, there are no reports related to the molecular activity of the peptide derived from hsa_circ_0006401. We found that hsa_circ_0006401 could promote an aggressive CRC phenotype by encoding hsa_circ_0006401 peptides. Many circRNA-derived proteins have sequence overlap with proteins conventionally generated from linear mRNA. Therefore, it is possible that hsa_circ_0006401-encoded proteins could interfere with the function of counterparts derived from linear mRNA COL6A3. COL6A3, encoded by the host gene *col6a3*, is an essential component of the extracellular matrix and structurally has a short triple-helical domain and two large globule-like N-terminal and C-terminal non-collagenous domains^18^. The cleaved C5 domain fragment, called endotrophin, can directly regulate the cancer phenotype by activating the TGFβ-dependent pathway^12^. Previous studies showed COL6A3 is a potential prognosis marker of colorectal carcinoma^19^ and alternatively spliced COL6A3 transcripts are associated with the progression of colon cancer^18,20^. In our study, we found COL6A3 is highly expressed in colon cancer, and its expression correlate with poor survival outcomes, which further emphasized the crucial role of COL6A3 as a tumor promoter in colorectal carcinoma. Moreover, hsa_circ_0006401 peptides decreased the mRNA and protein level of the host gene *col6a3* and TGFβ1 expression, the knockdown of hsa_circ_0006401 peptide also led to accelerated decay of the col6a3 mRNA, suggesting hsa_circ_0006401 peptides may promote proliferation and metastasis of CRC by protecting *col6a3* mRNA from degradation. Notably, hsa_circ_0006401 peptides were found to be closely related to poly(A) RNA binding and mRNA processing. The most important function of the poly A tail is to modulate the stability of mRNA as a complex^21^. Therefore, we speculated that hsa_circ_0006401 peptide may involve in the poly(A) mRNA decay process as RNA binding protein.

In conclusion, we revealed that hsa_circ_0006401 promotes proliferation and metastasis in vivo and in vitro by encoding a novel peptide, the peptide is essential and promotes stabilization of *col6a3* mRNA.
